# Swelling behaviours of compacted lime-softening sludge for application in landfill liners

**DOI:** 10.1038/s41598-021-94688-2

**Published:** 2021-07-27

**Authors:** Agnieszka Dąbska, Agata Léthel

**Affiliations:** 1 Faculty of Building Services, Hydro and Environmental Engineering, Warsaw University of Technology, 20 Nowowiejska Street, 00-653 Warsaw, Poland; 2 National Water Management Authority, 59A Żelazna Street, 00-848 Warsaw, Poland

**Keywords:** Environmental impact, Engineering

## Abstract

The objective of this study was to investigate the swelling potential of compacted lime-softening sludge for application in landfill liners. The study involved the assessment of the effect of compaction and moulding moisture content (30–40%), corresponding to the Proctor standard compaction test. One-dimensional oedometer swell tests were conducted using distilled water, tap water, and municipal landfill leachate, resulting in the determination of the expansion indices. Moreover, changes in the moisture content and dry density during the swelling process were investigated. The expansion index was significantly influenced by the initial moisture content and liquid chemistry. Subsequently, these factors also affected the sludge dry density decrease, and its moisture content increase, whereas the impact of the initial dry density on expansion was of low importance. An increase in the sludge moulding moisture content, limited swelling in all liquids used. The highest expansion, dry density, and moisture content changes due to swelling were identified for leachate at *w* < *w*_*opt*_. It should be underlined that the effect of liquid on the swelling potential faded away along with a further increase in the moisture content *w* > *w*_*opt*_. The novelty of the work lies in identifying a significant plunge of the expansion index at *w* ≈ *w*_*opt*_ for the leachate swelling test. The lime-softening sludge non-swelling moisture content was defined as *w*_*non*_ ≈ (*w*_*opt*_ + 4.0%) − (*w*_*opt*_ + 4.5%). For practical engineering implications, the moisture content between (*w*_*opt*_ + 2.0%) and (*w*_*opt*_ + 4.0%) was provided for the most suitable sludge application in landfill liners.

## Introduction

Lime-softening sludge is a by-product of the process of water treatment for industrial purposes. Water occurring in nature is frequently characterised by a high carbonate hardness caused by the presence of dissolved carbonates, bicarbonates, and calcium and magnesium hydroxides, namely, CaCO_3_, Ca(HCO_3_)_2_, Ca(OH)_2_, MgCO_3_, Mg(HCO_3_)_2_, and Mg(OH)_2_. The most applied method of carbonate hardness removal is water softening by means of adding lime Ca(OH)_2_^1^. The lime process of softening converts calcium and magnesium salts dissolved in water into precipitating insoluble compounds. The resulting calcium carbonate CaCO_3_ precipitates in the form of calcite, sometimes with an admixture of unstable forms, i.e., aragonite or vaterite, under lime-softening conditions. Magnesium hydroxide Mg(OH)_2_ precipitates solely as a gel sediment. Moreover, the lime-softening process results in the removal of suspensions and iron and manganese compounds^2^.

One of the methods for lime-softening sludge management is its application in geotechnics. Research on the geotechnical properties of lime-softening sludge was commenced by Glysson^3^, who considered the possibility of its reuse in landfill liners. Moreover, Glysson^3^ and Raghu and Hsieh^4^ proposed using lime-softening sludge in ground levelling and ground stabilisation in combination with other wastes. Lime-softening sludge has also been successfully applied in wastewater neutralisation^5^. Leeuwen et al.^6,7^ also demonstrated the potential use of dried lime sludge modified with stabilisers, or mixed with soil, Portland cement, or Class C fly ash as fill material for road construction. All these potential reuse options for lime-softening sludge were confirmed by Fei et al.^8^. Dąbska^9^ conducted a series of short-term tap water permeation tests of lime-softening sludge and long-term permeation tests using tap water, distilled water, NaOH solution with pH ≥ 11.0, HCl solution with pH ≤ 3.0, and municipal landfill leachate. The research confirmed that lime-softening sludge compacted at a moisture content higher than the optimum moisture content determined by the Proctor standard compaction test, i.e., *w*_*opt*_ + (1–2)% could replace mineral soils in landfill liners because it met the requirements of inert waste landfill liners ($k$≤ 1.0·10^−7^ m/s), irrespective of the leachate pH and hazardous and non-hazardous waste landfill sites (< 1.0·10^−9^ m/s). Regarding the material used for landfill liners, not only its permeation properties but also its bearing capacity, trafficability, internal and interface shear strength, compressibility, and resistance to desiccation cracking should be determined^10^. Furthermore, the swelling behaviour plays a determining role in the assessment of such properties, as unfavourable changes in geotechnical properties caused by the swelling process affect the permeability and mechanical properties. Hence, proper calculations for landfill liners require an assessment of the swelling properties^11–17^.

The lime-softening sludge geotechnical properties need to be specified in laboratory and filed tests before its usage in landfill liner. As sludge should be applied in layers, the manner in which the work is executed, such as the type of compactor, the thickness of the compacted layer and the number of passes of the machine on one track, should also be defined in the field^12^. The criterion of the hydraulic conductivity is the most important for landfill liners and it must be first met. The sludge compaction in the range of moisture contents between *w*_*opt*_ and *w*_*opt*_ + 4% at a dry density, which is not less than that indicated via the standard compaction Proctor test method of 0.95·ρ_ds_, allows to reach the lowest hydraulic conductivity and enables swelling reduction. The sludge should not be applied at the moisture content exceeding the plastic limit to avoid the problem of a low crack resistance that is too low and a shear strength that is too low^10,12^. The sludge compaction at a moisture content between *w*_*opt*_ + (1–2)% and approximately 41%, allows to achieve an internal friction angle between 36° and 38° and a cohesion of approximately 80–90 kPa. This should be performed by following the requirements of the hydraulic permeability, dry density, and shear strength^12^.

The swelling process occurs when soil is allowed free access to water upon unloading. As a result, an increase in the soil volume is observed. The swelling potential related microscale factors, such as the mineralogy, pore fluid chemistry, and soil structure, affect the density and water content. Consequently, these physical factors have an impact on the engineering properties of the soil^18^. Nonclay particles of the gravel, sand and most of the silt friction, out of which the most abundant mineral is quartz, are treated as inert. Their structures show high stability because of the lack of weakly bonded ions, which, in case of quartz that is made of silica tetrahedra, all tetrahedral oxygens bond to silicon. Thus, such minerals do not adsorb water and they are not swell-susceptible. In contrast, weak chemical bonds are observed in clay particles, such as montmorillonite, smectite, vermiculite or illite, allowing attraction and adsorption of water. This is because their structures also contain other layer silicates with structures made up of combinations of the silicon tetrahedron and the alumino-magnesium octahedron units. Weak bonds are characteristic of the oxygen atoms in the silicon tetrahedron and the hydroxyl atoms in the alumino-magnesium. Weak bonds allow the formation of a sheet structure in the octahedral units with shared hydroxyls. In the tetrahedron structure, the sheet structure with strong bonds is created at the base of the tetrahedron with adjacent tetrahedra, where the oxygen atoms are shared between the units. On the other hand, weak bonds are observed at apexes of the tetrahedron. Therefore, the silica sheets negative charges tend to be balanced by positively charged cations, balancing the repulsive electrical surface forces^13,18^. Water molecules attached on the particle surface, allow the swelling process to occur in the form of hydration. This stage is followed by an osmotic swelling phase, as water flows in the direction of the particle due to a gradient in the cation concentration between adsorbed water and pore water. An increasing distance from the particle surface causes the gradient’s decrease. The hydration and osmotic water, that are held close to the inner mineral core, create the double-layer adsorbed water. The water layer along with the adsorbed cations and the mineral create build the micelle, which constitutes an electrically neutral system due to the cations attracted by the negatively charged molecules^13^. The swelling process is affected by the interlayers or intersheets of minerals, the ion concentration, and the initial moisture content. The greater the mineral specific surface area and spacing between micelles are, the soil tends to be more swell-susceptible. An abundance of multivalent cations in the mineral structure significantly limits the expansive potential in comparison to that of the mineral with only monovalent cations^13,18–21^. Additionally, the susceptibility to swelling of structures with monovalent cations is much more strengthened by weak monovalent cation’s bonds than by those of divalent or trivalent cations^11^. Moreover, the lower the amount of water in pores, the greater the interaction between the micelles. The micelles are forced further apart during the swelling process as more water is adsorbed, causing an increase in the distance between particles and in the volume of pore spaces in soil structure^18–21^. For this reason, the soil pore pressure increases, leading to soil expansion in the volume. Subsequently, the swelling gradually slows down until it reaches equilibrium. As a consequence of the swelling process, the dry density decreases, and the moisture content increases^13,15,18–21^. Such parameter changes have a strong effect on the shear strength defined by an internal friction and a cohesion, and on the permeating parameter^11–16,22,23^. As a rule, the hydraulic conductivity reaches its minimum at a certain dry density in the specified range of the moisture content, located beyond the optimum moisture content. This hydraulic conductivity can only be achieved at that particular moisture content, when the distances between particles and the pore volume are the smallest. A further rise in the moisture content results in the hydraulic conductivity increase^13,24^. Moreover, the rise in distances between particles causes the cohesive forces to decrease^13,22,25^. Even if the cohesion is quite high at the initial moisture content, it can be reduced to almost zero at saturation state^26^. The moisture increase has a tendency to reduce the internal friction, as the increasing amount of water between particles reduces friction on their surfaces. Furthermore, the decrease of the density results in more free space around particles, easing their movement^15,22,23^. It should be noted that the swelling pressure is built up during the swelling process. If soil is not allowed to swell or the volume change of the soil is blocked, then the swelling pressure has an undesirable effect on an engineering structure. This can create additional pressure on the retaining structures and even, if it is high enough, lift the foundation^18,27^.

Hence, the following questions are in need of examination. What effect does the sludge moisture content and its dry density have on sludge swelling behaviours? Does the liquid type used in swell test contribute to the effect on the sludge swelling? What is the most suitable lime-softening sludge compaction due to its susceptibility to swelling for application in landfill liners?

The aim of this study was to investigate the swelling behaviour of lime-softening sludge, affected by the compaction and moulding moisture content in a one-dimensional oedometer swell test and the simultaneous liquid swell effect using distilled water, tap water, and municipal landfill leachate. In addition, the moisture content of the minimal sludge swelling potential was also assessed.

## Materials and methods

### Lime-softening sludge

The tested lime-softening sludge originated from the process of water treatment for cooling purposes with the application of a lime solution in the form of limewater at a concentration of 2%. Iron sulphate (FeSO_4_·7H_2_O) was applied in the coagulation process and was conducted in parallel to the lime-softening process. Sludge was directed to the filter press and removed to the container below after dewatering.

Based on the X-ray diffraction pattern, calcium carbonate (CaCO_3_) occurring in the form of calcite was identified as quantitatively dominant in the sludge composition. The differential thermal analysis performed simultaneously with the thermogravimetric analysis revealed that the calcium carbonate content was approximately 78.8%. This analysis also confirmed the presence of quartz in the sludge composition and did not exclude an insignificant content (up to 5.0%) of magnesium carbonate (MgCO_3_)^12^. The measurements of the sludge composition made by the carbonate bomb method pointed to the calcium carbonate content at a level of 83.3–87.3%. The content of silica (SiO_2_) was determined by the mineralisation method and ranged between 3.7 and 4.2%. The atomic absorption spectroscopy (AAS) procedure was used for the quantitative determination of chemical elements occurring in the sludge composition. The analysis showed the highest amounts of calcium (Ca^2+^) (31.27–32.26%), and relatively low concentrations of iron (Fe^3+^), magnesium (Mg^2+^), and aluminium (Al^3+^). Manganese (Mn^2+^), potassium (K^+^), titanium (Ti^2+^), and sodium (Na^+^) were revealed in trace amounts. The iron content in the sludge composition, which was higher than from other water treatment plants, was associated with the effect of coagulant FeSO_4_·7H_2_O. The sludge pH was 8.35–8.66. The chemical composition of lime-softening sludge is presented in Table 1.

**Table 1:** Chemical composition of lime-softening sludge^12^.

Parameter	Unit	Value
CaCO_3_	% by weight	83.3–87.3
SiO_2_		3.7–4.2
pH	–	8.35–8.66
Ca^2+^	% by weight	31.27–32.17
Fe^3+^		1.90–2.54
Mg^2+^		0.61–0.96
Al^3+^		0.11–0.16
Mn^2+^	ppm	284.6–478.0
K^+^		188.0–238.0
Ti^2+^		219.9–222.4
Na^+^		92.5–113.9

1 ppm = 1 mg/1 kg, 1% = 10,000 ppm.

**Figure 1:**
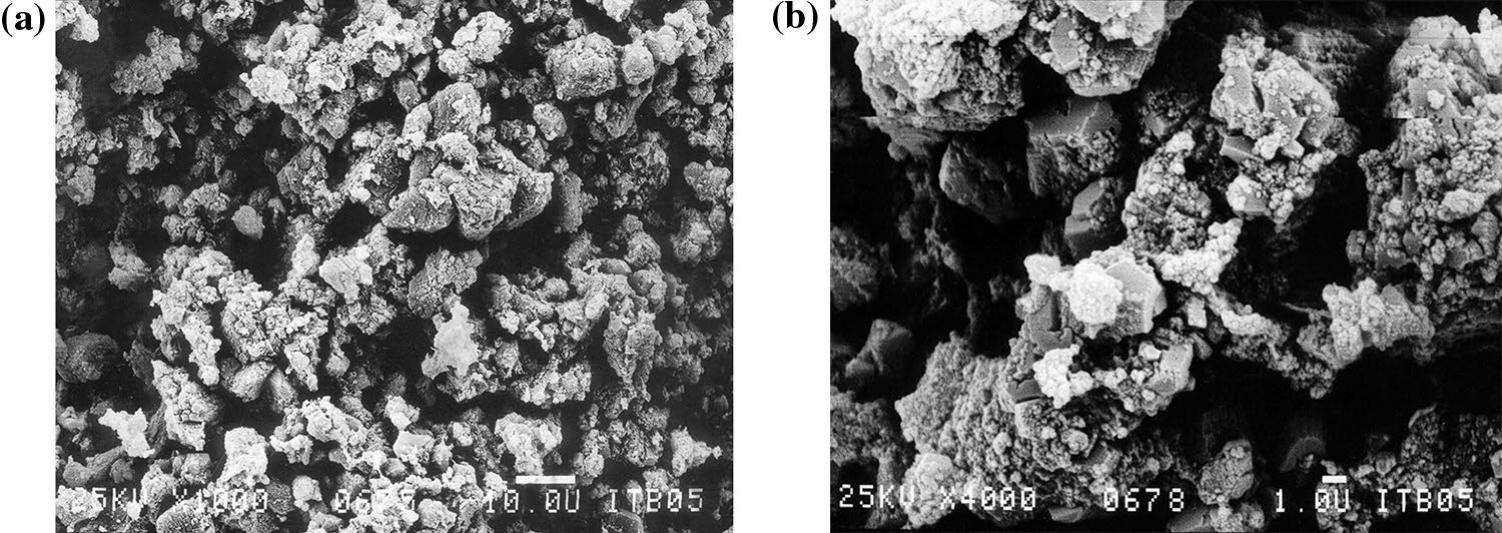
Crystalline structure of lime-softening sludge (SEM), magnified: (**a**) 1000 times and (**b**) 4000 times.

The X-ray diffraction analysis and scanning electron microscopy results confirmed the crystalline structure of the sludge, determining calcite as the form of calcium carbonate. Calcite developed compact aggregates with a size of approximately 10–20 μm, composed of finer crystals 1–2 μm in size, combined with even finer particles below 0.1 μm in size. The sludge structure is presented in Fig. 1.

In terms of the grain size, the analysed lime-softening sludge showed a similarity to silts and silty clays. The geotechnical properties of lime-softening sludge are presented in Table 2.

**Table 2:** Geotechnical properties of lime-softening sludge^9^.

No	Property	Value range	Remarks
1	Clay content (%)	Less than 23.7	
2	Silt content (%)	63.0–93.7	
3	Sand content (%)	Less than 22.0	
4	Bulk density $\rho$ (Mg/m^3^)	1.46–1.62	After filtering on filter press
5	Moisture content $w$ (%)	53.9–56.7	
7	Specific density of solids $\rho_{s}$ (Mg/m^3^)	2.72	
8	Shrinkage limit $w_{s}$ (%)	35.7	
9	Plastic limit $w_{p}$ (%)	37.8–41.1	
10	Liquid limit $w_{L}$ (%)	47.4	
11	Plasticity index $I_{p}$, (%)	6.3–9.6	
12	Activity $A_{c}$, (–)	0.57–0.87	According to Skempton^28^
13	Maximum dry density $\rho_{ds}$, (Mg/m^3^)	1.32	Proctor standard method
14	Optimum water content $w_{opt}$ (%)	36.1	

### Liquids used in the swell test

The swelling behaviour of lime-softening sludge was tested with free access to tap water, distilled water, and municipal landfill leachate from an operating landfill site in Otwock. Table 3 summarises the chemical properties of the liquids.

**Table 3:** Chemical properties of distilled water, tap water and leachate.

No	Parameter	Unit	Liquid		
			Distilled water	Tap water	Leachate
1	pH	–	7.80	7.70	8.35
2	Electric conduction	μS/cm	48.3	1513	12,615
3	ChZT	mgO_2_/dm^3^	–	–	3744
4	Ca^2+^	mgCa^2+^/dm^3^	0	59.0	42.9
5	Mg^2+^	mgMg^2+^/dm^3^	0	17.4	20.0
6	Na^+^	mgNa^+^/dm^3^	10.6	125.0	240.0
7	K^+^	mgK^+^/dm^3^	–	–	145
8	Fe^3+^	mgFe^2+^/dm^3^	0	0.057	8.24
9	Mn^2+^	mgMn^2+^/dm^3^	–	–	5.4
10	Cl^−^	mgCl^−^/dm^3^	21.3	259.0	2201
11	SO_4_^2−^	mgSO_4_^2−^/dm^3^	–	–	–
12	PO_4_^3−^	mgPO_4_^3−/^dm^3^	–	–	150
13	NH_4_^+^	mgNH_4_^+^/dm^3^	–	–	1226
14	NO_3_^−^	mgNO_3_^−^/Ndm^3^	–	–	–
15	N og	mgN/dm^3^	–	–	1305
16	N org	mgN/dm^3^	–	–	121
17	Pb^2+^	mgPb^2+^/dm^3^	–	–	0.504
18	Zn^2+^	mgZn^2+^/dm^3^	–	–	0.424
19	Cu^2+^	mgCu^2+/^dm^3^	–	–	624
20	Cr^2+^, Cr^7+^	mgCr^(7+)+(2+)^/dm^3^	–	–	0.392
21	Ni^2+^	mgNi^2+^/dm^3^	–	–	0.932
22	Cd^2+^	mgCd^2+^/dm^3^	–	–	0.088

### Sample preparation and testing procedure

The swell test was performed by the direct method as a one-dimensional oedometer test in accordance with procedure BS 1377-5^29^ and the guidelines^27,30^. The tests were carried out on cylindrical samples with a height of 0.01 m and diameter of 0.057 m, prepared in rigid-wall cylinders with filter paper placed at the bottom and top of the samples. The analysed sludge was dried to a dry state early, and then its moisture content was increased to a planned moulding water content by adding a proper amount of water. Hence, the sludge was kept in the tight container for a few days, which allowed the moisture in the sludge to distribute evenly throughout its mass. The moisture content was checked before sample preparation. Then, the sludge was compacted in a manual press directly in the cylinders in which it was tested. The samples were prepared to ensure that the dry densities *ρ*_*d*_ and moulding moisture contents $w$ corresponded with those from the compaction curve obtained from the Proctor compaction test to permit the determination of the expansion index and changes in the moisture content and dry density correlations depending on the initial compaction and moulding moisture content. The maximum dry density *ρ*_*ds*_ = 1.32 Mg/m^3^ and optimal moisture content *w*_*opt*_ = 36.1% were adopted on the basis of the compaction curve obtained using the Proctor standard method.

**Figure 2:**
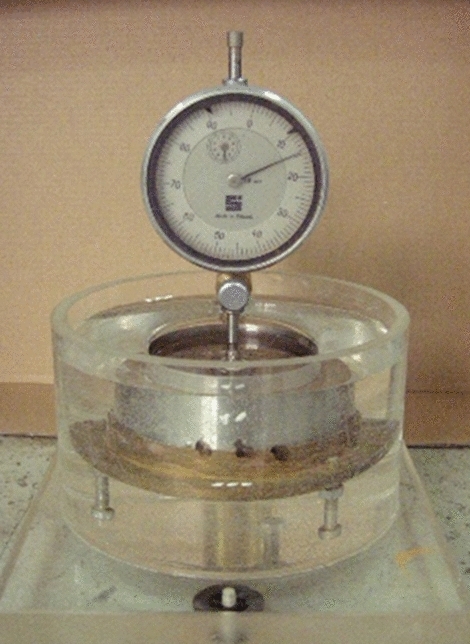
One-dimensional oedometer swell test in distilled water.

The samples were placed in the apparatus, and the dial indicators were installed in such a way that the increase in sample height could be observed (Fig. 2). Hence, the samples were flooded with liquids in a way that permitted the penetration of the samples from the bottom. The measurement of changes in the height of the samples commenced at the moment of their complete flooding. Readings were performed after 1, 2, 3, 4, 5, 10, 15, 30, 40, 50, and 60 min, with accuracy of 0.001 mm until equilibrium was established. It was assumed that swelling stabilisation occurred when no change in sample height was recorded for 10 min; i.e., two consecutive readings 10 min apart were the same.

The swell test was conducted for 3 liquids: distilled water, tap water, and municipal landfill leachate. The expansion index $EI$ was determined at a moulding moisture content $w$ of approximately 30%, 34%, 38%, and 40% for each liquid. As the tests were completed, the moisture content *w*_*f*_, bulk density *ρ*_*f*_, dry density *ρ*_*df*_, and void ratio *e*_*f*_ were determined. For each liquid and compaction, the test was conducted twice. A total of 24 tests were carried out. The results of all tests are shown as average values of the two measurements.

The expansion index $EI$ was evaluated as the ratio of a change in the sample height to its initial height and expressed in %, in accordance with the following equation^27,30^:

1 $${\text{EI}} = [(h_{1} - h_{0} )/h_{0} ] \cdot 100\%$$

where $EI$—expansion index, %, *h*_0_—initial sample height, m, *h*_1_—sample height after swelling, m.

## Results and discussion

### Swell tests results

**Figure 3:**
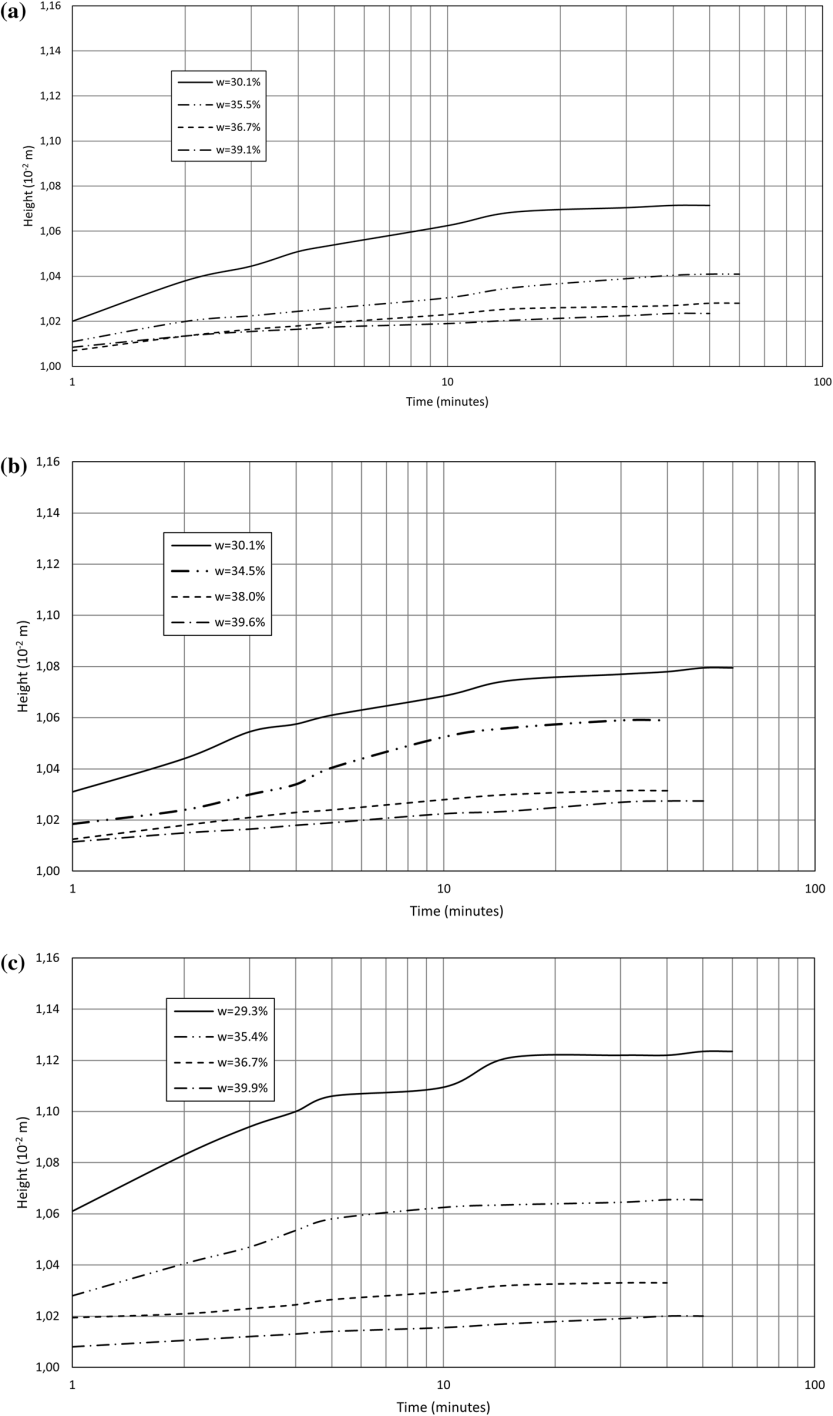
Sample height change versus log time for swell testing in (**a**) distilled water, (**b**) tap water, and (**c**) leachate.

The relations of the sample height change and log time for sludge swelling in distilled water, tap water, and leachate are presented in Fig. 3. The results of lime-softening sludge swell tests are summarised in Table 4. The swell time *t* in distilled water, tap water, and municipal landfill leachate ranges between 40 and 60 min, from an initial moisture content of 30% to 40%. No correlation is observed between the swell time *t* and the moulding moisture content *w*, dry density *ρ*_*d*_ (void ratio *e*), and any liquid type.

**Table 4:** Swell test results.

No	Bulk density $\rho$	Dry density $\rho_{d}$	Moulding moisture content $w$	Void ratio $e$	Expansion index $EI$	Final moisture content $w_{f}$	Final bulk density $\rho_{f}$	Final dry density $\rho_{df}$	Final void ratio $e_{f}$	Moisture content increase $\Delta w$	Dry density decrease $\Delta \rho_{d}$	Void ratio increase $\Delta e$	Time $t$
	(Mg/m^3^)	(Mg/m^3^)	(%)	(–)	(%)	(%)	(Mg/m^3^)	(Mg/m^3^)	(–)	(%)	(Mg/m^3^)	(–)	(min)
**Distilled water**													
1	1.62	1.25	30.1	1.18	7.1	48.9	1.71	1.15	1.37	18.8	0.10	0.19	50
2	1.80	1.33	35.5	1.05	4.1	44.4	1.76	1.22	1.24	8.9	0.11	0.18	60
3	1.80	1.31	36.7	1.07	2.8	42.2	1.76	1.23	1.20	5.5	0.08	0.13	60
4	1.78	1.28	39.1	1.12	2.3	40.5	1.79	1.27	1.14	1.4	0.01	0.02	50
**Tap water**													
5	1.62	1.25	30.1	1.18	7.9	48.4	1.72	1.16	1.35	18.3	0.09	0.17	60
6	1.78	1.33	34.5	1.05	5.9	44.2	1.72	1.20	1.28	9.7	0.13	0.22	40
7	1.79	1.30	38.0	1.09	3.1	43.5	1.75	1.22	1.23	5.5	0.08	0.14	60
8	1.79	1.29	39.6	1.12	2.7	42.0	1.77	1.24	1.19	2.4	0.04	0.07	50
**Leachate**													
9	1.60	1.24	29.3	1.20	12.4	50.5	1.71	1.14	1.39	21.2	0.10	0.19	60
10	1.79	1.32	35.4	1.06	6.6	48.1	1.73	1.17	1.33	12.7	0.15	0.27	50
11	1.79	1.31	36.7	1.07	3.3	43.1	1.78	1.24	1.19	6.4	0.07	0.11	40
12	1.78	1.28	39.9	1.13	2.0	41.6	1.78	1.26	1.16	1.7	0.02	0.03	50

### Dry density, moulding moisture content and liquid effects on the expansion index

**Figure 4:**
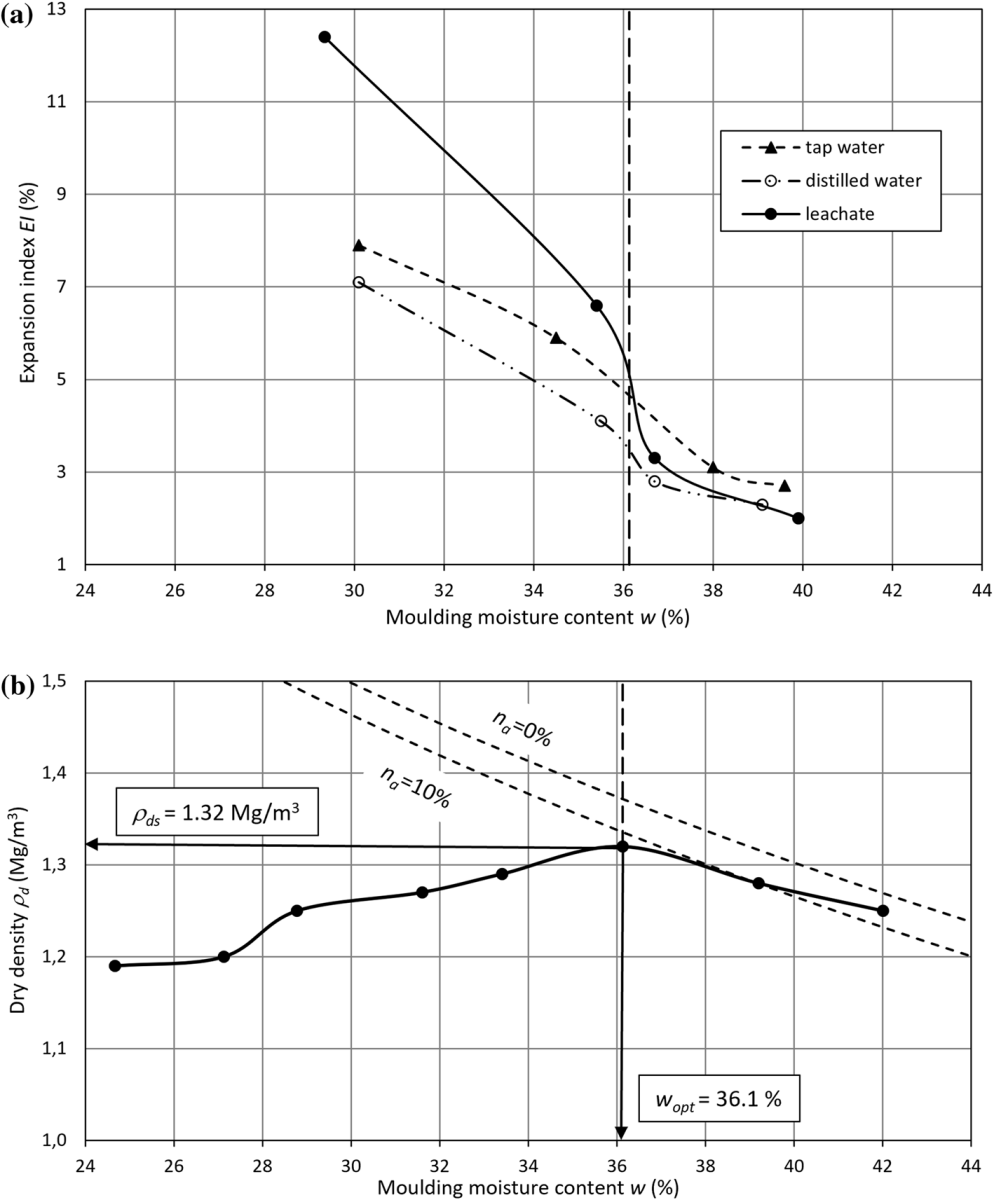
Expansion index versus (**a**) the moulding moisture content and (**b**) the dry density.

**Figure 5:**
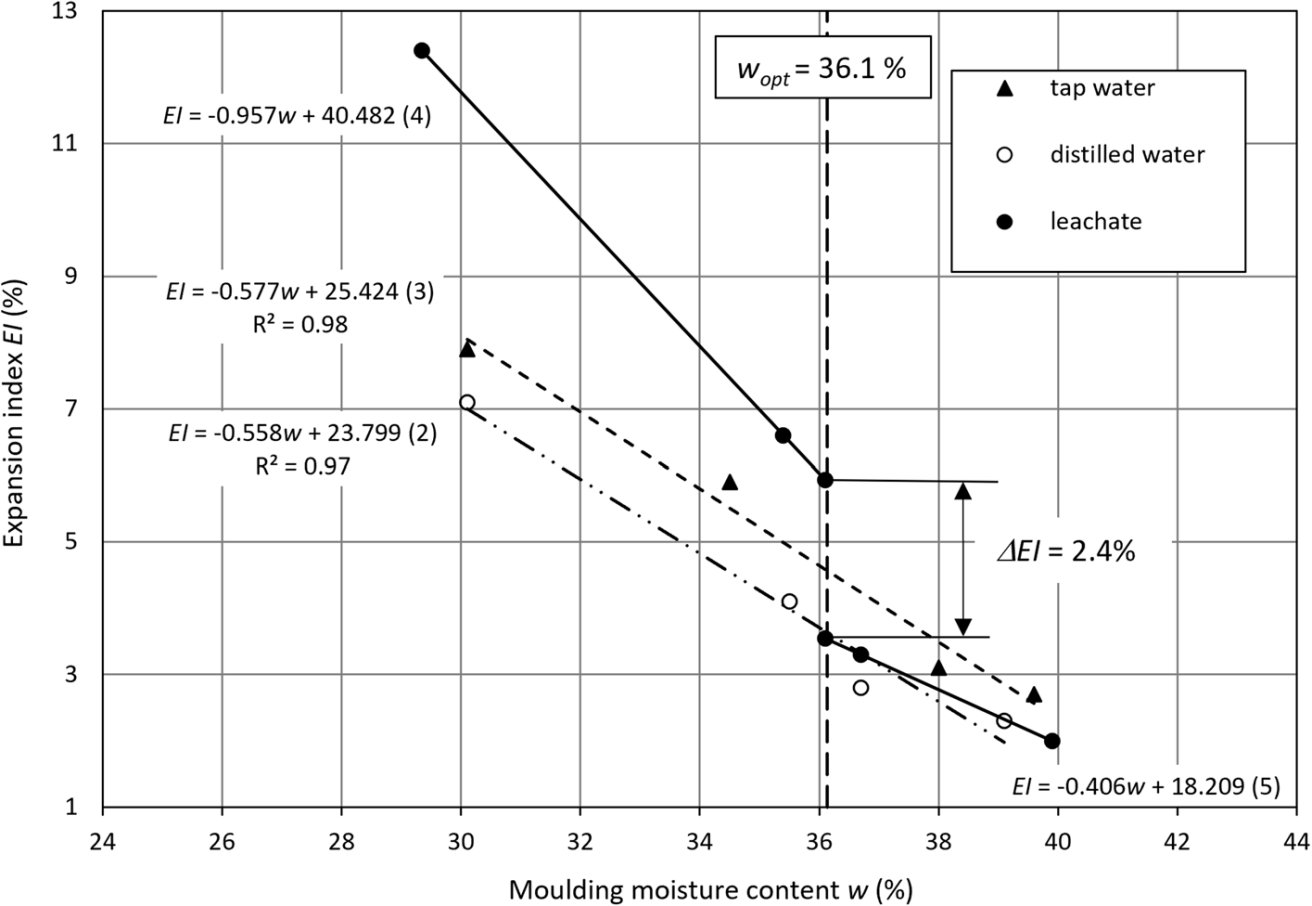
Correlation between the expansion index and moulding moisture content.

The expansion index $EI$ of lime-softening sludge at a moisture content between 30% ≈ *w*_*opt*_* − *6% and 40% ≈ *w*_*opt*_ + 4%, where the dry density (*ρ*_*d*_) and the moisture content (*w*) correspond to the compaction, obtained using the Proctor standard method, reach variable values. The range of variety depends on the moulding moisture content and liquid used for the test. The expansion index versus the dry density and moulding moisture content is presented in Fig. 4. For all types of liquid, the expansion index of lime-softening sludge shows a downward trend with an increase in its moulding moisture content at an initial rise and then a decrease in the dry density. Such a dependency suggests that the swelling properties of sludge are affected more by its moulding moisture content than by its compaction. At a moisture content of 30%, the lowest value of the expansion index is obtained for swelling in distilled water (7.1%), which is inconsiderably higher in tap water (7.9%) and the highest in leachate (12.4%). The greatest decrease in the expansion index is observed for leachate, followed by tap water and distilled water with a moisture content from 30 to 40%. This index is reduced by $\Delta EI$ = 10.4%, $\Delta EI$ = 5.2% and $\Delta EI$ = 4.8%. The dependency of the expansion index on the moulding moisture content can be calculated with a decreasing linear function (Fig. 5):

2 $${\text{In}}\;{\text{distilled}}\;{\text{water}}:\quad EI = - 0.558w + 23.799\quad R^{2} = \, 0.{97 }\left( \% \right)$$

3 $${\text{In}}\;{\text{tap}}\;{\text{water}}:\quad EI = - 0.577w + 25.424\quad R^{2} = 0.{98}\left( \% \right)$$

The values of the expansion index in distilled water are approximately 1% lower than those for swelling in tap water throughout the entire range of moisture content. For leachate, the expansion index plummets at *w* ≈ *w*_*opt*_. The measurement points are located on straight lines described by the following correlations (Fig. 5):

4 $${\text{In}}\;{\text{leachate}}\;{\text{at}}\;w < w_{opt} :\quad EI = - 0.957w + 40.482\left( \% \right)$$

5 $${\text{In}}\;{\text{leachate}}\;{\text{at}}\;w > w_{opt} :\quad EI = - 0.406w + {18}.{2}0{9 }\left( \% \right)$$

There is a jump in the expansion index by $\Delta EI$ = 2.4% at *w* ≈ *w*_*opt*_ when estimated from Eqs. (4) and (5). The expansion index decreases to 2% at a moisture content of 40% for swelling in the leachate. There is little disparity between the leachate results and the distilled and tap water results at 40%.

There is abundant evidence that swelling of sludge moulded at a moisture content *w* < *w*_*opt*_ is greater than that of sludge moulded at *w* > *w*_*opt*_. This finding results from different sludge structures, which, similar to cohesive soils, depends on the moulding moisture content^18,19^. The structure influences the swelling that is initiated by a reduction in the effective stress by the addition of water^13^. Particles are arranged in different directions at a moisture content *w* < *w*_*opt*_. These particles form an irregular structure in which only part of the pores are filled with water and hardly any connections of bound water films exist. Subsequently, free space occurs between particles, which is later filled with liquid in which the material, subject to a swell test, is placed. Hence, there is still a potential to attract and attach water molecules onto the particle surface. In contrast, particle assemblages are arranged in a regular way at the moisture content *w* > *w*_*opt*_. Moreover, at the moisture content *w* > *w*_*opt*_, free water fills a considerably greater volume of pores compared to the moisture content *w* < *w*_*opt*_, i.e., 90% or more. On the wet side of the compaction curve, there are many more connections of bound water films between micelles, which have a lower potential to attract water. Therefore, permeating the liquid into the pores of such a structure as well as ion exchange on the particle surface are limited. Hence, lower values of the expansion index are obtained for a material with the same compaction at the moisture content *w* > *w*_*opt*_ compared with *w* < *w*_*opt*_. The moulding moisture content of compacted lime-softening sludge has a pronounced effect on its swell. Consequently, the swelling behaviour of compacted lime-softening sludge follows the rules of expansive soils. The drier the initial state of the soil is, the greater the expansion potential of the soil^15,31–35^.

The expansion index of lime-softening sludge experiences a higher value in leachate than in tap water, followed by distilled water at the moisture content *w* < *w*_*opt*_. This behaviour is likely associated with the chemical composition of liquids. That is, the higher the pH value is, the more types of salt there are and the higher their concentration is, the greater the lime-softening sludge swelling. It is clearly visible that leachate supplies more multivalent cations than distilled water and tap water (Table 3). These cations can replace monovalent cations present in sludge particles. The obtained results seem to be similar to those received by Nayak et al.^36^ and Shariatmadari et al.^37^, who found that an increase in the leachate concentration caused a rise in the void ratio and soil volume; however, a higher concentration of electrolytes in liquid causes a decrease in the adsorbed water layer thickness, limiting swelling in natural soils^13,18,38,39^. The swelling behaviours of lime-softening sludge in leachate might be directly related to the concentration of sodium ions in the liquid and the precipitation of CaCO_3_ in sludge^40^. In addition, the results of swell tests can also be influenced by the liquid pH, as it is established that a rise in cation exchange is affected by the pH; i.e., cation exchange significantly decreased below pH < 7^20,40^. Thus, the liquid pH value impacts the swelling potential of lime-softening sludge more than the concentration of cations compared to natural soils at *w* < *w*_*opt*_. However, further, more detailed research concentrated on liquid chemistry and its changes during the swelling process is needed.

The liquid chemistry is of paramount importance for the swelling behaviour of lime-softening sludge at the moisture content *w* < *w*_*opt*_, whereas the effect of the liquid type on sludge swelling declines on the wet side of the compaction curve (*w* > *w*_*opt*_). This decrease results from the lower ability of micelles to attract water, as their adsorbed water layers make more connections, so the micelles are closer to reaching their ion-exchange capacity. The contact of the permeating liquid with particles, caused by free water filling nearly the entire pore volume, is also limited. A plunge of the expansion index in the leachate should be associated with a lack of permeating liquid access to the particle surface. This decrease is a consequence of the formation of a regular structure with particles entirely surrounded by bound water films at *w* ≈ *w*_*opt*_, i.e., a limited possibility of ion exchange. Due to the high swelling potential of sludge in the leachate in comparison to swelling in distilled water and tap water, a sharp limitation in the possibility of ion exchange is observed in the form of an abrupt decline in the expansion index.

### Dry density and moisture content changes

**Figure 6:**
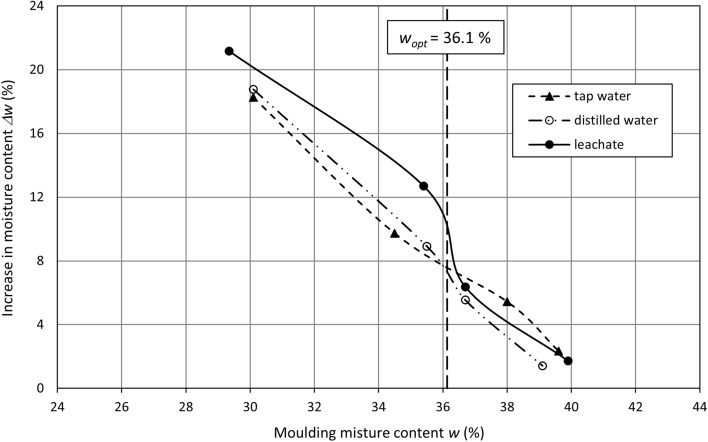
Moisture content increase versus moulding moisture content.

**Figure 7:**
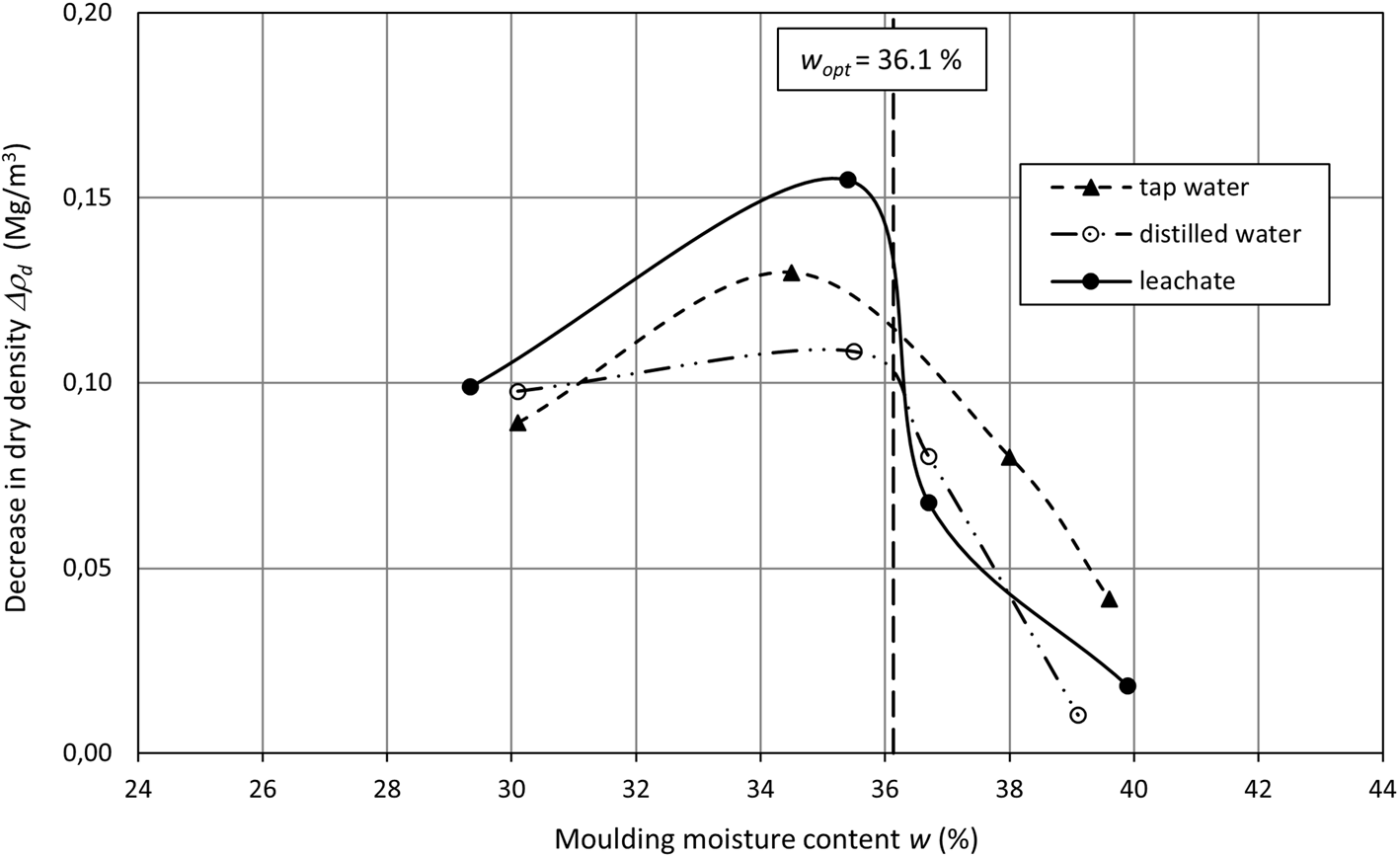
Dry density decrease versus moulding moisture content.

Lime-softening sludge subjected to swelling shows an increase in the moisture content and a decrease in the dry density for all liquids. The dry density changes correspond to the void ratio changes, as the void ratio rises while the dry density decreases (Table 4). Changes in the moisture content Δ*w* and dry density Δ*ρ*_*d*_ depending on the moulding moisture content are presented in Figs. 6 and 7, respectively.

The dependency of changes in the sludge moisture content during swelling on its moulding moisture content shows a similar behaviour to that of the expansion index, i.e., changes in the sample volume. An increase in the moisture content falls between an initial moisture content of 30% and 40%. The highest increase in the moisture content is determined at the lowest moulding moisture content, and it is approximately 18% for swelling in tap water and distilled water and approximately 21% for swelling in the leachate (Fig. 6). The difference in the increases in the moisture content is comparable throughout the entire moulding moisture range for distilled water and tap water and reaches approximately 1%. For distilled water, the increase in the moisture content is insignificantly higher on the dry side of the Proctor curve (*w* < *w*_*opt*_), whereas it is slightly lower on the wet side of the compaction curve (*w* > *w*_*opt*_). The samples that swell in leachate are characterised by the highest rise in the moisture content. A moisture content increase takes rapidly declines at *w* ≈ *w*_*opt*_. At the moulding moisture content *w* > *w*_*opt*_, the growth in the moisture content in leachate is comparable with that in distilled water and tap water; the difference between them is very small. The sludge experiences a moisture increase of approximately 2% in all liquids at a moulding moisture content of 40%. The increase in the moisture content reaches approximately 60% for distilled water and tap water and over 70% for leachate in relation to the moulding moisture content of 30%; it is reduced to 4–5% at an initial moisture content of 40%, reaching 15–17% when exceeding the optimum moisture content.

There is a decrease in the dry density for swelling in all liquids between moisture contents of 30% and 40% (Fig. 7). The decrease in dry density rises steadily from approximately 0.10 Mg/m^3^ (void radio equal approximately 0.19) and reaches the highest point at a moisture content that is inconsiderably lower than the optimum moisture content (*w* ≤ *w*_*opt*_) for all liquids. The highest dry density decrease varies from approximately 0.11 Mg/m^3^ for distilled water to approximately 0.15 Mg/m^3^ for leachate, constituting 8% and 12% of the initial dry density, respectively. Whereas the highest void ratio increase corresponds to the dry density changes. It varies from approximately 0.19 for distilled water to approximately 0.27 for leachate, constituting 16% and 25% of the void ratio, respectively. From that moisture content, the trend reverses. The changes in the dry density decrease gradually for distilled and tap water, whereas in the case of the leachate, a visible decline is observed from 0.15 Mg/m^3^ (*w* ≤ *w*_*opt*_) to 0.07 Mg/m^3^ (*w* ≥ *w*_*opt*_). A decrease in the dry density is 0.01–0.04 Mg/m^3^ at a moisture content of 40%, which corresponds to an increase in void ratio 0.02–0.07.

The characteristics of the moisture content and dry density changes resulting from the swelling of lime-softening sludge are similar to those observed for natural soils swelling in distilled water and tap water^15,31,41^. This results from the sludge structure, as in the case of the expansion index. At a lower moisture content, the bound water films are thinner, with hardly any connections between them, and the particles still have the potential to adsorb water molecules until equilibrium swelling is established. The lower the initial moisture content is, i.e. the greater the sludge swelling potential is, the higher the increase in the moisture content due to swelling. In contrast, the highest decrease in the dry density occurs at *w* ≤ *w*_*opt*_, i.e., at *ρ*_*d*_ ≤ *ρ*_*ds,*_ which is the maximum compaction at which liquid penetration and ion exchange on the particle surface is still possible^15,31–35^. Thus, an initial dry density influences its reduction. Additionally, the dry density is also affected by the sludge moulding moisture, as different decreases in the density are observed for almost the same compaction.

### Non-swelling moisture content

**Figure 8:**
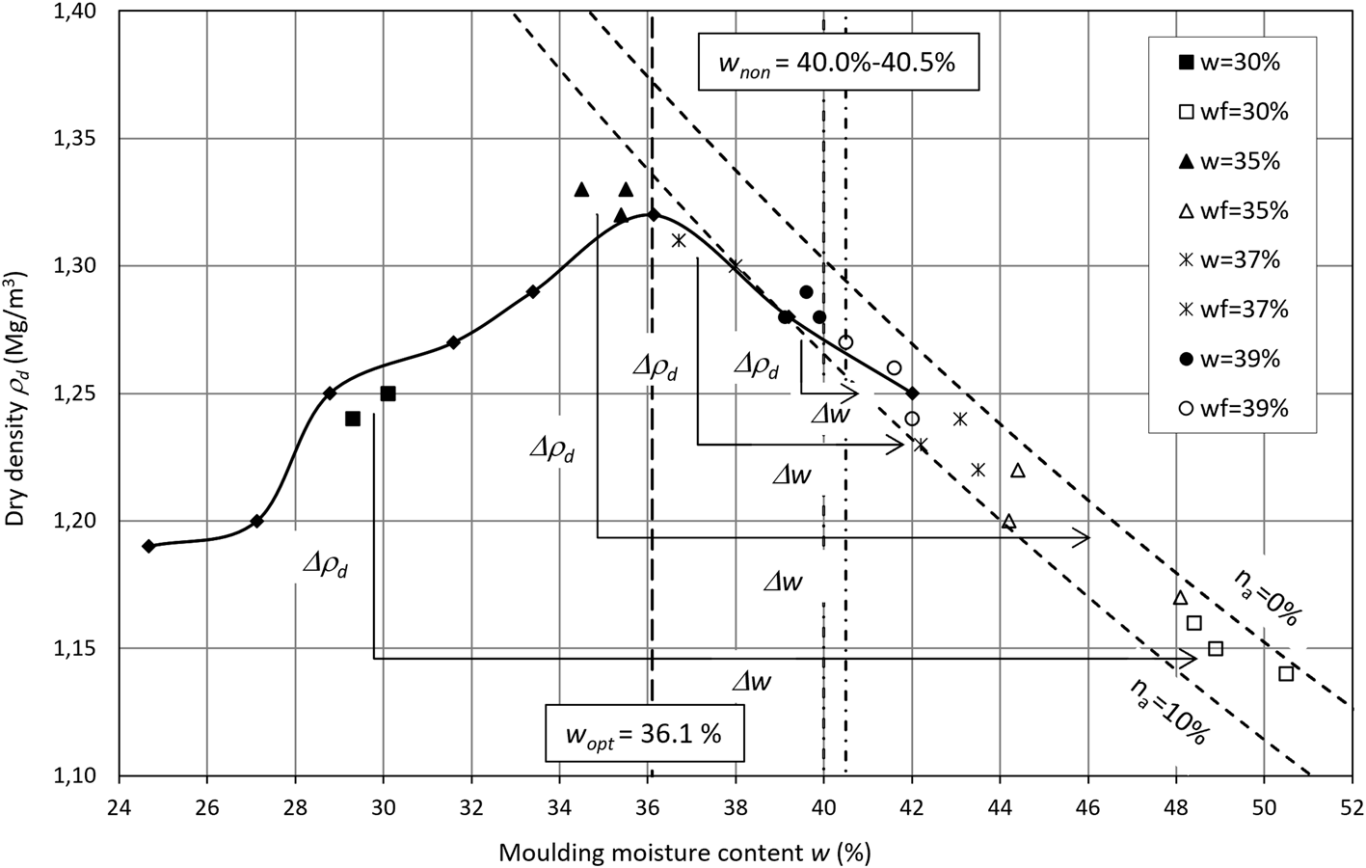
Moisture content and dry density changes in relation to the Proctor compaction curve.

Changes in the moisture content and dry density of lime-softening sludge during the swelling process suggest that the moisture content at which the swelling process practically does not occur can be defined (Fig. 8). The moisture content of the minimal sludge swelling potential can be referred to as the non-swelling moisture content. This content is associated with reaching the sludge ion-exchange capacity and then establishing the equilibrium of swelling. The lime-softening sludge non-swelling moisture content is *w*_*non*_ $\approx$ 40.0–40.5% ≈ (*w*_*opt*_ + 4.0%) − (*w*_*opt*_ + 4.5%). This moisture content corresponds to a dry density approximately 1.27 Mg/m^3^, i.e., *ρ*_*d*_ ≈ 0.96*ρ*_*ds*_.

### Swelling potential of lime-softening sludge

Many criteria resulting from both indirect and direct methods are available for the identification of the swelling potential of natural soils^42^. Based on the analysis of indirect criteria referring to the physical properties of lime-softening sludge, i.e., the liquid limit, plasticity index, shrinkage limit, and percent clay size fraction (*w*_*L*_ = 47.7%, *I*_*p*_ = 6.3–9.6%, *w*_*s*_ = 35.7%, < 0.002 mm = 11%), the sludge swelling potential can be classified as low (*I*_*p*_ = 0–15% and *I*_*p*_ < 18%, *w*_*s*_ > 15%, *w*_*L*_ < 50% and the percent clay size fraction < 30%)^19,26,43,44^ or high (*w*_*L*_ = 40–60%)^43^. By analysing its activity, lime-softening sludge can be described as inactive/normal (*A*_*c*_ < 1.25)^28^. According to the expansion index criterion based on the one-dimensional oedometer test, the degree of lime-softening sludge expansivity is low, as most expansion index values are lower than 10%^27,41^. There is an exception to sludge swelling in leachate at moulding moisture contents lower than approximately 32% ($EI >$ 10%). Hence, the swelling potential can be classified as medium. The attempt to classify the swelling potential of lime-softening sludge shows that the shrinkage limit cannot be successfully used to predict its swelling behaviour. It can be concluded that indirect methods based on physical properties do not always permit a proper estimation of the lime-softening sludge swelling properties and can lead to an incorrect assessment of its swelling potential.

The low swelling potential of lime-softening sludge should be associated with the mineral composition of its solid phase, as the content of clay minerals is supposed to be relatively low because the clay content is less than 23.7%. The sludge solid phase is mostly composed of calcite with a relatively small specific surface area and composition of exchangeable cations. The sorption complex of the sludge primarily contains divalent (Ca^2+^, Mg^2+^) and trivalent cations (Fe^3+^), characterised by limited swelling in comparison to univalent cations. Univalent cations (Na^+^, K^+^), which are easily exchangeable, occur in trace amounts in the sludge composition (Table 1)^11,18,20^.

## Conclusions

This paper examines the swelling behaviour of lime-softening sludge and the effects of compaction and liquid chemistry on its expansion index. Swell tests were performed on compacted samples using distilled water, tap water, and municipal landfill leachate. All tests were conducted in a laboratory; thus, the results should be confirmed in the field tests before implementing the sludge in landfill liner. Based on the study results, the following conclusions were drawn:

1. The direct methods, i.e., one-dimensional oedometer swell tests should be implemented to assess the lime-softening sludge swelling potential.
2. The lime-softening sludge expansion index value of up to 10% indicates a low degree of swelling. The relatively small specific surface area of calcite and the high number of divalent cations in the sludge composition can be responsible for the sludge low swell-susceptibility. However, sludge affected by municipal landfill leachate at a moisture content lower than approximately 32%, ($EI$ > 10%) is an exception resulting from leachate chemistry.
3. The factors of the sludge moulding moisture content and liquid chemistry decisively affected the sludge swell-susceptibility and the changes in its physical properties. The dry density fulfils a secondary role in shaping the sludge swelling potential.
4. An increase in the initial moisture content and a decrease in ion concentration in liquids significantly limits the sludge swelling potential. The study establishes the linear correlation of expansion indices and moisture content between *w*_*opt*_-6% and *w*_*opt*_ + 4% for all liquids. The new discovery is that the index declines when swelling in leachate at a moisture content of *w* ≈ *w*_*opt*_. This decline might be expounded by the more complex chemical composition of leachate, richer in divalent and trivalent cations, compared to water.
5. A decrease in the sludge dry density and an increase in its moisture content are observed, irrespective of the applied liquid type. The highest values of the expansion index, and the changes in the moisture content and the dry density are recorded for leachate at moulding moisture contents *w* < *w*_*opt*_, while the liquid type effect on these values is not observed at *w* > *w*_*opt.*_. This finding points to the diminishing swell-susceptibility of lime-softening sludge and the limited influence of liquid type on its swelling properties.
6. The defined lime-softening sludge non-swelling moisture content *w*_*non*_ ≈ (*w*_*opt*_ + 4.0%) − (*w*_*opt*_ + 4.5%) corresponds to the dry density *ρ*_*d*_ ≈ 0.96*ρ*_*ds*_. The moulding moisture content of sludge between (*w*_*opt*_ + 2.0%) and (*w*_*opt*_ + 4.0%) is provided for the most appropriate and stable time properties of sludge, ensuring the safe work of waste landfill liners in landfills due to the lime-softening sludge hydraulic permeability, mechanical parameters, and swelling potential.
7. While the effect of the moulding moisture content and compaction on the swelling behaviours of lime-softening sludge seems to be sufficiently recognised, the liquid chemistry impact on sludge swelling requires wider and more detailed investigation in further stages of research.
